# A minimum assumption approach to MEG sensor array design

**DOI:** 10.1088/1361-6560/ace306

**Published:** 2023-08-23

**Authors:** Andrey Zhdanov, Jussi Nurminen, Joonas Iivanainen, Samu Taulu

**Affiliations:** 1 BioMag Laboratory, HUS Diagnostic Center, Helsinki University Hospital and University of Helsinki, Helsinki, Finland; 2 Department of Physics, University of Washington, Seattle, WA, United States of America; 3 Motion Analysis Laboratory, Children’s Hospital, University of Helsinki and Helsinki University Hospital, Helsinki, Finland; 4 Sandia National Laboratories, Albuquerque, NM 87185, United States of America; 5 Institute for Learning and Brain Sciences, University of Washington, Seattle, WA, United States of America

**Keywords:** MEG, vector spherical harmonics, optimization, sensor array, optically pumped magnetometer, channel information capacity

## Abstract

*Objective.* Our objective is to formulate the problem of the magnetoencephalographic (MEG) sensor array design as a well-posed engineering problem of accurately measuring the neuronal magnetic fields. This is in contrast to the traditional approach that formulates the sensor array design problem in terms of neurobiological interpretability the sensor array measurements. *Approach.* We use the vector spherical harmonics (VSH) formalism to define a figure-of-merit for an MEG sensor array. We start with an observation that, under certain reasonable assumptions, any array of *m* perfectly noiseless sensors will attain exactly the same performance, regardless of the sensors’ locations and orientations (with the exception of a negligible set of singularly bad sensor configurations). We proceed to the conclusion that under the aforementioned assumptions, the only difference between different array configurations is the effect of (sensor) noise on their performance. We then propose a figure-of-merit that quantifies, with a single number, how much the sensor array in question amplifies the sensor noise. *Main results.* We derive a formula for intuitively meaningful, yet mathematically rigorous figure-of-merit that summarizes how desirable a particular sensor array design is. We demonstrate that this figure-of-merit is well-behaved enough to be used as a cost function for a general-purpose nonlinear optimization methods such as simulated annealing. We also show that sensor array configurations obtained by such optimizations exhibit properties that are typically expected of ‘high-quality’ MEG sensor arrays, e.g. high channel information capacity. *Significance.* Our work paves the way toward designing better MEG sensor arrays by isolating the engineering problem of measuring the neuromagnetic fields out of the bigger problem of studying brain function through neuromagnetic measurements.

## 1. Introduction

Magnetoencephalography (MEG) is a noninvasive brain imaging modality that studies neuronal activity through measurement, outside of the head, of magnetic fields created by neuronal currents (Hämäläinen *et al* 1993, Cohen and Halgren 2009). Electric currents in the brain (intracranial currents), in accordance with Maxwell’s equations, produce magnetic fields that extend to the volume outside the head, where they can be measured noninvasively. In MEG, one measures the magnetic fields with sensors located outside the head and tries to infer the intracranial currents generating these measurements. The fact that the magnetic fields outside the head (extracranial magnetic fields) are related to intracranial currents through Maxwell’s equations makes it possible to infer, with limited certainty, spatiotemporal features of the intracranial currents from the signals measured with MEG sensors.

Ideally, we would like to measure the extracranial fields and compute the intracranial currents that produced them. Such computation is called *the inverse problem*. However, in the general case, the inverse problem is *ill-posed*—it cannot be solved uniquely. This is because some intracranial currents produce zero extracranial magnetic field.^7^ 
^7^
This statement needs a bit of explanation. An important property of the quasistatic Maxwell equations is their linearity—the magnetic fields are related to the intensity of the currents that cause them by a linear transformation. Any linear transformation has this property: it is possible to uniquely identify the transformation’s input given its output (i.e. compute the inverse transformation) if there exists no non-zero input that causes the transformation to produce zero output. That is why the impossibility to uniquely estimate the intracranial currents from the extracranial fields is the same thing as existence of a non-zero intracranial current that produces zero extracranial magnetic field. One particularly celebrated example of ‘a silent current’—radial current dipole in a spherically symmetric conductor—is described in Sarvas (1987). Obviously, no extracranial MEG measurement can reveal anything about the silent currents.

Whereas no MEG sensor array (collection of magnetic field sensors) located outside the head can reveal everything about the intracranial currents, some sensor arrays can reveal much more than others, depending on the number, locations, orientations of sensors and other parameters (Kemppainen and Ilmoniemi 1989, Hämäläinen *et al* 1993).

There is another complication to the problem of MEG measurement, a more practical one. So far we assumed that the extracranial magnetic fields are caused by the intracranial currents only. However, in any practical situation the measurements of magnetic fields around the head will be contaminated by environmental noise (magnetic fields produced by various artificial sources: power grid, elevators, electric motors operating nearby, etc). The environmental noise can be, to a considerable degree, reduced by various shielding techniques (e.g. Taulu *et al* 2019), however in any practical MEG setup the residual noise is still non-negligible. Thus the magnetic fields measured by the sensors are a sum of two components: (a) the neuronal component—the magnetic fields produced by the intracranial currents and associated volume currents in the head, and (b) the environmental noise produced by much stronger currents located far away from the sensors. We would like our sensor array to reveal as much as possible about the the intracranial currents not only in the noiseless case, but also in the presence of the environmental noise.

All of the above makes MEG sensor array design an important problem that has attracted considerable attention. One very tempting approach to MEG sensor array design is to try to summarize the ‘goodness’ of the array using a single scalar—figure-of-merit. Once we find a figure-of-merit that describes well enough the array’s ability to characterize intracranial currents (preferably, in the presence of environmental noise), sensor array design becomes a multidimensional nonlinear optimization problem—a problem that has been widely studied, and for which multiple practical tools are available. Unsurprisingly, a variety of figures-of-merit have been proposed to date. These include measures such as precision in locating cortical current sources (Hämäläinen *et al* 1993, Beltrachini *et al* 2021), and information about the sources conveyed by the array (Kemppainen and Ilmoniemi 1989, Schneiderman 2014, Iivanainen *et al* 2021).

As we mentioned before, the ultimate, albeit unreachable (in the general case) goal of MEG is to solve the inverse problem. Therefore, it is not surprising that some of the figures-of-merit proposed to date: (a) make some assumptions about all possible intracranial currents that improve the conditioning of the inverse problem, and (b) summarize in a single number the sensor array’s ability to solve the inverse problem under these assumptions. The problem with this approach is that it critically depends on the accuracy of the assumptions, but there is no good way to ensure such accuracy. Additionally, previously proposed figures-of-merit generally focus on the sensor array’s performance in the absence of environmental noise (Kemppainen and Ilmoniemi 1989, Hämäläinen *et al* 1993).

In the current paper we propose a novel figure-of-merit for MEG sensor array design that is not centered around solving the inverse problem. We do not try to solve an ill-posed problem of characterizing the intracranial currents through additional assumptions that improve the conditioning. Instead, following the approach by Ahonen *et al* (1993), Grover and Venkatesh (2016), Iivanainen *et al* (2021), we solve the much less ambitious, but well-conditioned problem of measuring the magnetic fields outside of the head as accurately as possible. This approach might seem counterintuitive as it explicitly ignores the inverse problem and instead focuses on measuring as accurately as possible something that might be of no interest (per se) to MEG users—the magnetic field outside the head. Nonetheless, we argue that separating the question ‘What can we say about intracranial currents from extracranial magnetic field measurements?’ from the question ‘How can we measure extracranial magnetic fields as accurately as possible?’ makes a lot of sense from the sensor array designer’s perspective.^8^ 
^8^
Strictly speaking, we are cheating here a little bit. When we talk about ‘…measuring …magnetic fields as accurately as possible’ we implicitly assume that there exists some way to define what ‘accurate’ means—in other words, that there is a way to measure the (dis-)similarity between the two magnetic fields (the true one and the estimate) as a single scalar. In reality there are many different ways to quantify the difference between two magnetic fields, each leading to a different definition for figure-of-merit of the sensor array. In our simulations we used the *L* ^∞^-norm to define the difference, but other other choices are equally possible. The former question necessarily requires some assumptions about the intracranial currents, which are particularly problematic during the array design stage since these assumptions are specific to a particular MEG experiment. The latter question, on the other hand, is independent of such assumptions and constitutes exactly the question that MEG sensor array designer should address.

As an additional benefit, our approach provides a straightforward and principled way of incorporating the resilience to the environmental noise into the figure-of-merit. The question ‘How can we measure extracranial magnetic fields as accurately as possible?’ can be naturally extended to the question ‘How can we measure the neuronal component of the extracranial fields as accurately as possible?’ without the need for arbitrary weight factors balancing the accuracy of the inverse problem solution against the noise resilience.

Briefly stated, our approach consists of using the vector spherical harmonics (VSH) decomposition (Hill 1954, Taulu and Kajola 2005) of the magnetic field to define a field model which we use to optimize the sensor array. Using the VSH decomposition we define cutoff values for the spherical harmonics degrees *l* of the inner and outer expansions corresponding to fields due to neural sources and external interference, respectively. By using the VSH field model, we investigate how measurement noise maps into magnetic field interpolation noise for a given sensor array configuration. We define a figure of merit that quantifies how much the noise gets amplified in the process. We design sensor arrays that minimize the figure of merit, i.e, that aim not to amplify noise.

## 2. Methods

### 2.1. Array geometry

#### 2.1.1. Array geometry constraints

When designing an MEG sensor array, we cannot place the sensors completely freely. For example, we cannot place them inside the head, or too far away from the head, or too close to each other, etc. We denote the set of all admissible sensor configurations as **Ξ**. Each point *ξ* ∈ **Ξ** is a possible sensor array; **Ξ** is the domain of the sensor array optimization problem.

For the purpose of this paper, we assume point-like sensors that measure magnetic field along a certain direction (sensor orientation). There are no constraints on sensor orientations; the only constraint on sensor locations is that all the sensors are located within a closed volume adjacent to the head, called *sampling volume* *V*_samp_. Thus **Ξ** is uniquely defined by *V*_samp_ and the number of sensors *m*

\begin{eqnarray*}{\boldsymbol{\Xi }}\triangleq {\{({\bf{r}},{\bf{e}})| {\bf{r}}\in {V}_{\mathrm{samp}},\parallel {\bf{e}}\parallel =1\}}^{m},\end{eqnarray*}

where **r** and **e** denote the location and orientation of a sensor, respectively. Whereas these assumptions are not perfectly realistic, the resulting simulations provide important insights into the real-world MEG sensor array design as we will see in section 3.

**Figure 1.**
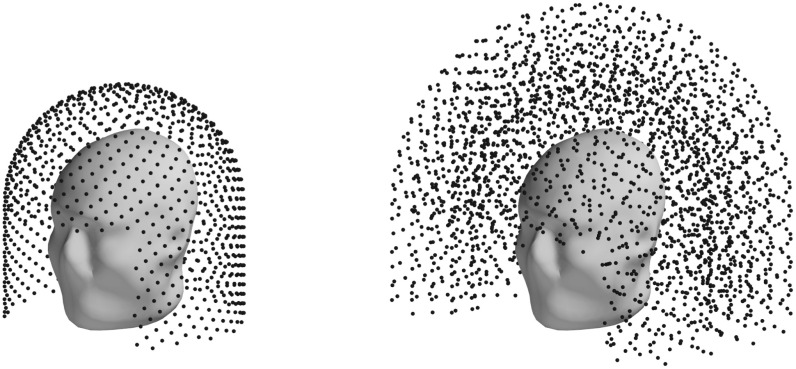
Sampling volume used in our paper. Blue dots are sampling locations used for the discretization of the continuous sampling volume {**r**∣**r** ∈ *V*_samp_}. For 2D (left) and 3D (right) sampling volumes.

In this paper we mostly consider two different sampling volumes: a 3D and a 2D. Both are helmet-shaped, defined as a union of two geometric primitives (see figure 1):

1. A section of a cylindrical shell (wrapped around the subject’s head with an opening in front of the face), and
2. A hemispherical shell covering the top of the head

The height of the cylindrical shell was 15 cm, and the opening spanned an angle of *π*/2 radian. For the 3D volume both primitives have a finite thickness of 0.1 m (inner radius of 0.15 m and outer radius of 0.25 m), and for the 2D volume both have zero thickness with the inner and outer radii being equal to some value *R* (and to each other). Thus for the 2D sampling volume our sensor array is a function of *R*.^9^ 
^9^
Note that *R* affects only the radii of the two primitives comprising the sampling volume, whereas the height of the cylindrical shell is fixed to 0.15 m independently of *R*. Thus arrays for different values of *R* are not scaled versions of each other.


**Figure 2.**
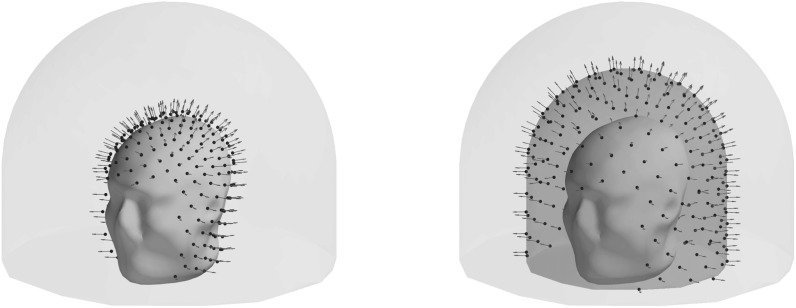
The difference between the anatomically-constrained (left) and regular (right) 3D sampling volumes. In both figures the sensors are located on the innermost wall of the sampling volume. Blue spheres mark sensor locations; red arrows denote the direction along which the magnetic field is measured.

In addition to the 3D sampling volume described above, in some of our experiments we also use an anatomically-constrained variation of the 3D sampling volume. The main difference between the two is that in the anatomically-constrained version the inner wall of the sampling volume is defined by the subject’s individual anatomy—sensors can be placed anywhere down to the distance of 7 mm from the MRI-based anatomical head surface (see figure 2). The offset of 7 mm is based on a typical dimension of an OPM sensor (Shah *et al* 2018); for the purpose of our simulations we used an anatomical head surface provided by the MNE-Python package (Gramfort *et al* 2013).

Note that whereas 3D sampling volume is a closed region of 3D space of non-zero volume, the 2D volume is a zero-volume surface. Nevertheless, we will use the term ‘volume’ for both of them for convenience.

#### 2.1.2. Uniformly-spaced radial arrays

We define a special type of MEG sensor array—an (approximately) uniformly-spaced radial array—that we are going to use as an example of what a reasonable non-optimized MEG sensor array might look like.

**Figure 3.**
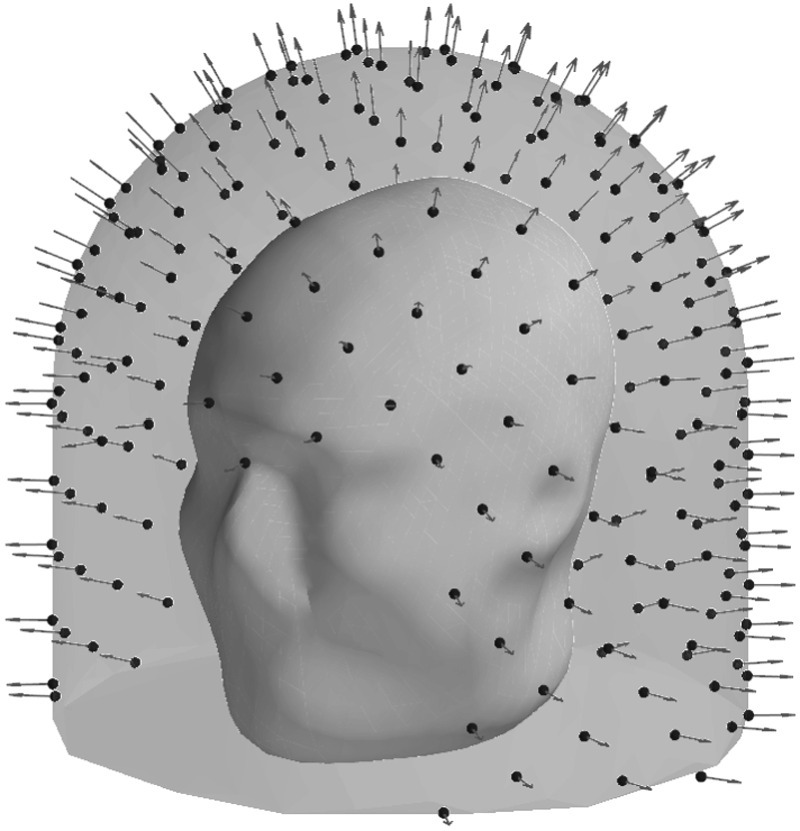
A uniformly-spaced radial sensor array. Blue spheres mark sensor locations; red arrows denote the direction along which the magnetic field is measured.

The uniformly-spaced radial array of *N* sensors (see figure 3) comprises *N* sensors approximately uniformly distributed over the 2D sampling volume of radius *R*. The points are distributed over the sampling volume (2D surface in this case) using an algorithm based on the idea of the generalized spiral set on a sphere (Saff and Kuijlaars 1997). The orientations of the sensors (e.g. the directions along which the magnetic field is measured) are normal to the sampling volume. Thus uniformly-spaced radial array is a function of *N* and *R*.

### 2.2. Array figure-of-merit definition

Let us assume that we are given a sampling volume *V*_samp_ and number of sensors *m*, thus defining the domain **Ξ** of our sensor array optimization problem. For each particular sensor array configuration *ξ* ∈ **Ξ** we have *m* measurements of the magnetic field at *m* (possibly distinct) locations within *V*_samp_. At each location **r** we measure a single component of the magnetic field vector **B**(**r**), along the particular sensor’s orientation. We assume that everywhere throughout *V*_samp_ the value of **B**(**r**) is accurately enough approximated by the first *n* VSH components (where *n* = *L*_*α*_(*L*_*α*_ + 2) + *L*_*β*_(*L*_*β*_ + 2) for some appropriately chosen positive integers *L*_*α*_ and *L*_*β*_):^10^ 
^10^
For more details on this, see the supplementary material.

\begin{eqnarray*}\begin{array}{rcl}{\bf{B}}({\bf{r}}) &amp; = &amp; {{\bf{B}}}_{\alpha }({\bf{r}})+{{\bf{B}}}_{\beta }({\bf{r}})=\displaystyle \sum _{l=1}^{{L}_{\alpha }}\displaystyle \sum _{m=-l}^{l}{\alpha }_{{lm}}{{\bf{B}}}_{{\alpha }_{{lm}}}({\bf{r}})\\ &amp; &amp; +\displaystyle \sum _{l=1}^{{L}_{\beta }}\displaystyle \sum _{m=-1}^{l}{\beta }_{{lm}}{{\bf{B}}}_{{\beta }_{{lm}}}({\bf{r}}),\end{array}\end{eqnarray*}

where ${{\bf{B}}}_{{\alpha }_{{lm}}}({\bf{r}})$ and ${{\bf{B}}}_{{\beta }_{{lm}}}({\bf{r}})$ are VSH basis functions that are perfectly known, representing neuromagnetic and interference field components, respectively, *α*_*lm*_ and *β*_*lm*_ are the unknown VSH coefficients that depend on the distribution of the intracranial currents and the environmental noise sources, respectively. Then, in the notation of Taulu and Kajola (2005), our measurement constitutes a linear operator given by a VSH matrix **S**:

\begin{eqnarray*}{\boldsymbol{\phi }}={\bf{Sx}},\end{eqnarray*}

where ***ϕ*** is the vector of values measured by the MEG sensor array, **x** is the vector of the VSH coefficients

\begin{eqnarray*}{\bf{x}}={\left[{\alpha }_{1,-1},\ldots ,{\alpha }_{{L}_{\alpha },{L}_{\alpha }},{\beta }_{1,-1},\ldots ,{\beta }_{{L}_{\beta },{L}_{\beta }}\right]}^{T},\end{eqnarray*}

and **S** is an *m* × *n* matrix determined by the sensor array geometry, where *m* is the number of sensors and *n* is the number of VSH components.

Note that the VSH basis allows us to separate the neuronal fields from the environmental noise. We can write **x** as a sum of **x**_*α*_ and **x**_*β*_, containing coefficients for the internal and external parts of the VSH expansion:

\begin{eqnarray*}\begin{array}{l}{\bf{x}}={{\bf{x}}}_{\alpha }+{{\bf{x}}}_{\beta }\\ {{\bf{x}}}_{\alpha }={{\bf{I}}}_{\alpha }{\bf{x}}\\ {{\bf{x}}}_{\beta }={{\bf{I}}}_{\beta }{\bf{x}}.\end{array}\end{eqnarray*}

Here **I**_*α*_ and **I**_*β*_ are diagonal selector matrices that respectively select only internal or external basis coefficients from **x**

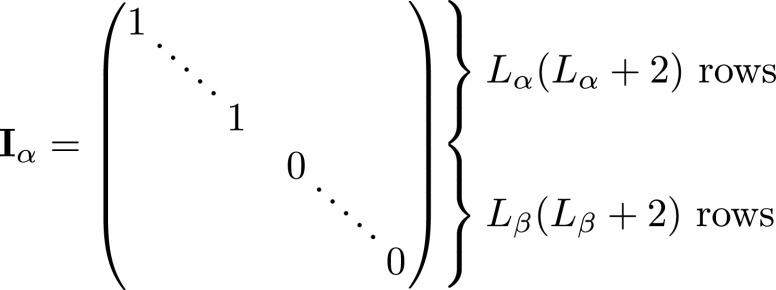
and
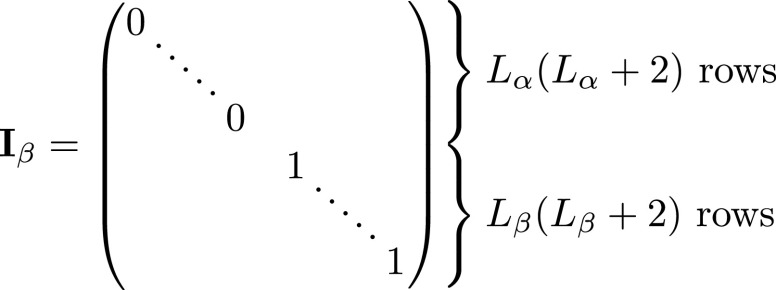



Assuming that *m* ≥ *n* and the rank of **S** is *n*, we can solve equation (3) for **x**:

\begin{eqnarray*}{\bf{x}}={{\bf{S}}}^{\dagger }{\boldsymbol{\phi }},\end{eqnarray*}

where **S** ^†^ is the Moore–Penrose pseudoinverse of **S**. Now, let us consider some *possible* sensor location (and orientation, as we assume that our sensor only measures the magnetic field along its orientation), where we *could have* placed the sensor. For any possible location **r** ∈ *V*_samp_ and orientation **e** (**e** is a unit vector) the reading of the sensor *ϕ*(**r**, **e**) would be:

\begin{eqnarray*}\phi ({\bf{r}},{\bf{e}})={{\bf{s}}}_{{\bf{r}},{\bf{e}}}{\bf{x}}={{\bf{s}}}_{{\bf{r}},{\bf{e}}}{{\bf{S}}}^{\dagger }{\boldsymbol{\phi }},\end{eqnarray*}

where **s**_**r**,**e**_ is a row vector of length *n* specifying the values of the VSH components at (**r**, **e**). Equation (5) is essentially an interpolation procedure that allows us to compute the readings of any possible sensor located anywhere in the sampling volume.

Now, let us go one step further and say that we want to estimate only the neuronal component *ϕ*_*α*_(**r**, **e**) of the possible measurement *ϕ*(**r**, **e**), without the environmental noise. To achieve this we restrict the interpolation to the inner basis only:

\begin{eqnarray*}{\phi }_{\alpha }({\bf{r}},{\bf{e}})={{\bf{s}}}_{{\bf{r}},{\bf{e}}}{{\bf{x}}}_{\alpha }={{\bf{s}}}_{{\bf{r}},{\bf{e}}}{{\bf{I}}}_{\alpha }{{\bf{S}}}^{\dagger }{\boldsymbol{\phi }}.\end{eqnarray*}

Equation (6) holds exactly if the measurements ***ϕ*** are exact and all the assumptions outlined above (namely, that the first *n* VSH components capture all the energy of the magnetic field and **S** is full rank) hold. In this case, it does not matter where our sensors are located, we will always be able to perfectly simulate *any* sensor array restricted to the sampling volume.

However, in reality the sensors are noisy. Thus, instead of reading true values of magnetic field ***ϕ***, the sensors give us a noisy estimate $\hat{{\boldsymbol{\phi }}}$

\begin{eqnarray*}\hat{{\boldsymbol{\phi }}}={\boldsymbol{\phi }}+{{\boldsymbol{\phi }}}_{\mathrm{noise}}.\end{eqnarray*}

Substituting equation (7) into (6) gives us the noise for the estimation of the *ϕ*_*α*_, which we call the interpolation noise:^11^ 
^11^
Note that computing *ϕ*_*α*_ involves not only interpolation, but also external noise rejection.

\begin{eqnarray*}\begin{array}{rcl}{{\bf{s}}}_{{\bf{r}},{\bf{e}}}{{\bf{I}}}_{\alpha }{{\bf{S}}}^{\dagger }\hat{{\boldsymbol{\phi }}} &amp; = &amp; {{\bf{s}}}_{{\bf{r}},{\bf{e}}}{{\bf{I}}}_{\alpha }{{\bf{S}}}^{\dagger }({\boldsymbol{\phi }}+{{\boldsymbol{\phi }}}_{\mathrm{noise}})\\ &amp; = &amp; {{\bf{s}}}_{{\bf{r}},{\bf{e}}}{{\bf{I}}}_{\alpha }{{\bf{S}}}^{\dagger }{\boldsymbol{\phi }}+{{\bf{s}}}_{{\bf{r}},{\bf{e}}}{{\bf{I}}}_{\alpha }{{\bf{S}}}^{\dagger }{{\boldsymbol{\phi }}}_{\mathrm{noise}}\\ &amp; = &amp; {\phi }_{\alpha }({\bf{r}},{\bf{e}})+{{\bf{s}}}_{{\bf{r}},{\bf{e}}}{{\bf{I}}}_{\alpha }{{\bf{S}}}^{\dagger }{{\boldsymbol{\phi }}}_{\mathrm{noise}}\\ &amp; = &amp; {\phi }_{\alpha }({\bf{r}},{\bf{e}})+{\phi }_{\mathrm{noise}}({\bf{r}},{\bf{e}}).\end{array}\end{eqnarray*}

If we define our sensor array configuration by a vector ***ξ*** that contains all sensors’ locations and orientations, and observe that **S** ^†^ is a function of ***ξ***, we will see that each sensor array configuration ***ξ*** yields an interpolation noise distribution over the sampling volume:

\begin{eqnarray*}{\phi }_{\mathrm{noise}}({\bf{r}},{\bf{e}},{\boldsymbol{\xi }})={{\bf{s}}}_{{\bf{r}},{\bf{e}}}{{\bf{I}}}_{\alpha }{{\bf{S}}}^{\dagger }({\boldsymbol{\xi }}){{\boldsymbol{\phi }}}_{\mathrm{noise}}.\end{eqnarray*}

Observe that the term **s**_**r**,**e**_ **I**_*α*_ **S** ^†^(***ξ***) in equation (9) is a row vector of the length *m* (number of sensors). We assume that sensor noise ***ϕ***_noise_ is a Gaussian, zero-mean random vector with the elements independent and identically distributed, where each component has variance of *σ* ^2^ ^12^ 
^12^
This is quite a reasonable assumption for real-world MEG devices. Note, however, that our analysis can be trivially extended to the more general case where each sensor has a different noise variance.. Then *ϕ*_noise_(**r**, **e**, ***ξ***) becomes a zero-mean Gaussian random variable with variance ∥**s**_**r**,**e**_ **I**_*α*_ **S** ^†^(***ξ***)∥^2^ *σ* ^2^, where ∥•∥ denotes the Frobenius norm of a vector. Hence, for each sensor array configuration ***ξ*** and each location-orientation pair (**r**, **e**):

\begin{eqnarray*}{\phi }_{\mathrm{noise}}({\bf{r}},{\bf{e}},{\boldsymbol{\xi }})\sim { \mathcal N }(0,{\sigma }_{\mathrm{interp}}{\left({\bf{r}},{\bf{e}},{\boldsymbol{\xi }}\right)}^{2}),\end{eqnarray*}

where

\begin{eqnarray*}{\sigma }_{\mathrm{interp}}({\bf{r}},{\bf{e}},{\boldsymbol{\xi }})=\parallel {{\bf{s}}}_{{\bf{r}},{\bf{e}}}{{\bf{I}}}_{\alpha }{{\bf{S}}}^{\dagger }({\boldsymbol{\xi }})\parallel \sigma =\parallel {{\bf{s}}}_{{\bf{r}},{\bf{e}}}{{\bf{I}}}_{\alpha }{\left({\bf{S}}{\left({\boldsymbol{\xi }}\right)}^{T}{\bf{S}}({\boldsymbol{\xi }})\right)}^{-1}{\bf{S}}{\left({\boldsymbol{\xi }}\right)}^{T}\parallel \sigma .\end{eqnarray*}

*σ*_interp_(**r**, **e**, ***ξ***) describes the distribution of noise *ϕ*_noise_ at each location-orientation point (**r**, **e**). We want to summarize the spatial distribution of *σ*_interp_(**r**, **e**, ***ξ***) over the whole sampling volume with a single value that will serve as a figure-of-merit for comparing different sensor arrays. There are numerous ways to do this; for the purpose of this paper we define the figure-of-merit to be the maximum of $\tfrac{{\sigma }_{\mathrm{interp}}({\bf{r}},{\bf{e}},{\boldsymbol{\xi }})}{\sigma }$ over the sampling volume:

\begin{eqnarray*}\begin{array}{rcl}q({\boldsymbol{\xi }}) &amp; \triangleq &amp; \mathop{\max }\limits_{\displaystyle \genfrac{}{}{0em}{}{{\bf{r}}\in {V}_{\mathrm{samp}}}{\parallel {\bf{e}}\parallel =1}}\displaystyle \frac{{\sigma }_{\mathrm{interp}}({\bf{r}},{\bf{e}},{\boldsymbol{\xi }})}{\sigma }\\ &amp; = &amp; \mathop{\max }\limits_{\displaystyle \genfrac{}{}{0em}{}{{\bf{r}}\in {V}_{\mathrm{samp}}}{\parallel {\bf{e}}\parallel =1}}\parallel {{\bf{s}}}_{{\bf{r}},{\bf{e}}}{{\bf{I}}}_{\alpha }{{\bf{S}}}^{\dagger }({\boldsymbol{\xi }})\parallel \\ &amp; = &amp; \mathop{\max }\limits_{\displaystyle \genfrac{}{}{0em}{}{{\bf{r}}\in {V}_{\mathrm{samp}}}{\parallel {\bf{e}}\parallel =1}}\parallel {{\bf{s}}}_{{\bf{r}},{\bf{e}}}{{\bf{I}}}_{\alpha }{\left({\bf{S}}{\left({\boldsymbol{\xi }}\right)}^{T}{\bf{S}}({\boldsymbol{\xi }})\right)}^{-1}{\bf{S}}{\left({\boldsymbol{\xi }}\right)}^{T}\parallel .\end{array}\end{eqnarray*}

Intuitively, one can think about *q*(***ξ***) in the following way: assume I have an array ***ξ*** of sensors with additive gaussian noise of variance *σ* ^2^. Throughout the array’s sampling volume I have a magnetic field that is a sum of two components: the brain magnetic field (signal of interest) and the environmental magnetic field (interference). If my sensor array has the figure-of-merit value of *q*, it means that I will be able to estimate the signal-of-interest component of the field anywhere within the sampling volume; my estimate will be noisy with additive gaussian noise of standard deviation not worse than *q* *σ*. One can think of *q* as the worst-case noise amplification factor; we are going to use the term ‘noise amplification factor’ throughout the paper.

Once the figure-of-merit *q*(***ξ***) is defined, finding the best sensor array ***ξ***_opt_ becomes an optimization problem

\begin{eqnarray*}{{\boldsymbol{\xi }}}_{\mathrm{opt}}=\mathop{\mathrm{argmin}}\limits_{{\boldsymbol{\xi }}\in {\boldsymbol{\Xi }}}q({\boldsymbol{\xi }}),\end{eqnarray*}

where **Ξ** is the domain of the optimization problem defined by the sampling volume and the number of sensors *m* (for the definition of **Ξ** see equation (1)).

In this paper we try to solve equation (13) using numerical nonlinear optimization algorithms. We report the improvement in *q*(***ξ***) yielded by the optimization, demonstrate the resulting sensor geometries, and compare our figure-of-merit to the information-capacity-based figure-of-merit proposed in the previous works.

### 2.3. Channel information capacity of a sensor array

We wanted to compare the behavior of our proposed figure-of-merit to some established metric that has been used by the MEG community. We chose channel information capacity (Kemppainen and Ilmoniemi 1989) as a reference metric for such a comparison. Channel information capacity measures the amount of information (quantified as number of bits per sample) that the magnetic field as measured by the array conveys about the distribution of current sources inside the head, under particular assumptions about the source distribution and its statistics.

Under the assumption of spatial white sensor noise with variance *σ* ^2^ and a Gaussian source distribution with a covariance matrix **Σ**, the information capacity can be calculated as (Kemppainen and Ilmoniemi 1989)

\begin{eqnarray*}I=\displaystyle \frac{1}{2}\displaystyle \sum _{i}{\mathrm{log}}_{2}({P}_{i}+1)=\displaystyle \frac{1}{2}\displaystyle \sum _{i}{\mathrm{log}}_{2}\left(\displaystyle \frac{{\lambda }_{i}^{2}}{{\sigma }^{2}}+1\right),\end{eqnarray*}

where *P*_*i*_ are the SNRs of the orthogonal channels defined by the eigencomponents and the eigenvalues *λ*_*i*_ of the covariance matrix **L** **Σ** **L** ^*T*^, where **L** is the lead-field matrix representing the measured magnetic fields produced by the sources.

### 2.4. Implementation details

We did all the computations in Python 3 programming language using popular libraries for scientific computing and visualization such as SciPy (Virtanen *et al* 2020), NumPy (Harris *et al* 2020), and mayavi (Ramachandran and Varoquaux 2011). All the source code used for the simulations described in this paper is available from GitHub (Zhdanov and Nurminen 2023) under the GNU General Public License (Gnu general public license 2007).

#### 2.4.1. VSH computation

We computed **S**(***ξ***) and **s**_**r**,**e**_ using the implementation of VSHs in MNE-Python (Gramfort *et al* 2013). We used *L*_*α*_ = 10 and *L*_*β*_ = 3 for the VSH expansion, which resulted in 135 components in the expansion.

#### 2.4.2. Approximating spatial distribution of interpolation noise

Theoretically, *q*(***ξ***) is defined as a maximum of a continuous function ∥**s**_**r**,**e**_ **I**_*α*_ **S** ^†^(***ξ***)∥ over a bounded domain {(**r**, **e**)∣**r** ∈ *V*_samp_, ∥**e**∥ = 1} = {**r**∣**r** ∈ *V*_samp_} × {**e**∣∥**e**∥ = 1} (see equation (12)). In practice, we approximated the continuous domain {**r**∣**r** ∈ *V*_samp_} by a dense discrete grid of 2500 points for the 3D and 1000 points for the 2D sampling volumes.

For the 2D sampling volume, the 1000 points are approximately uniformly spread across the helmet surface^13^ 
^13^
We distribute the points on the helmet surface are using a variation of the ‘golden ratio’ algorithm for approximately evenly distributing points on a spherical surface. (see figure 1 left). Helmet surface being approximately 0.25 m^2^, the resulting density of the sampling locations is about 1 location per 0.000 25 m^2^.

For the 3D sampling volume, the sampling grid comprises 5 concentric shells, each shell similar to the 2D volume described above. The shells radii are uniformly distributed on the interval 0.15–0.25 m, making the radial spacing between two neighboring shells 0.02 m. Each shell has 500 sampling locations uniformly distributed across it, for the outermost shell this leads to the density of about 1 sampling location per 0.001 m^2^.

#### 2.4.3. Initialization of the optimization procedure

The optimization procedure is initialized with a uniformly-spaced radial sensor configuration (see section uniformly-spaced radial arrays for more details). For the 3D array optimization procedure, we try three different initial conditions corresponding the radii *R* = 0.15 m, *R* = 0.2 m, and *R* = 0.25 m for the initial sensor array configuration.

#### 2.4.4. Optimization procedure

We evaluated several general-purpose nonlinear optimization algorithms: basin-hopping, differential evolution, and dual annealing. Of these, the dual annealing demonstrated the best performance, so we used it for all the work described in the paper.

The dual annealing, as implemented by the scipy.optimize.dual_annealing function of the scipy toolbox, is a stochastic optimization algorithm derived from the generalized simulated annealing (Xiang *et al* 1997). This method combines the classical simulated annealing with the fast simulated annealing algorithms augmented by a local search on accepted locations.

We used the default values for the maximum number of global iterations (1000) and the limit for the number of objective function calls (10^7^). These parameters resulted in a optimization run lasting 3–5 d on a typical desktop computer.

It is important to note, that dual annealing is an optimization procedure over a continuous parameter space: the parameter variables are not restricted to a set of possible discrete values. The only constraint that we used during the optimization process was the requirement that all the sensors should be inside the sampling volume.

#### 2.4.5. Channel information capacity computation

We used 1000 random current dipoles to compute the channel information capacity. Each dipole’s location was randomly chosen from a uniform distribution from a spherical volume of radius 0.07 m centered at the origin. Each dipole’s orientation was randomly chosen from a uniform distribution on a sphere. The total dipole moment (root-sum-squared across all the dipoles) was 2 × 10^−8^ A m and the standard deviation of the sensor noise was 10^−14^ T.

### 2.5. Computational experiments

In this paper we report the results of three computational experiments.

#### 2.5.1. Investigation of uniformly-spaced radial arrays

As a uniformly-shaped radial sensor array is a function of its radius *R* and the number of sensors *N*, in our first computational experiment we study the behavior of the array’s noise amplification factor as a function of these two parameters.

#### 2.5.2. Array optimization based on a 3D sampling volume

In the second experiment we try to find an optimal (w.r.t. the noise amplification factor) design for a sensor array of 240 sensors within a 3D sampling volume. We investigate the stabilty of the optimization procedure w.r.t. the starting condition by running multiple experiments with different initial conditions.

Additionally, we investigate the behavior of the optimization procedure for different orders of the expansion of the VSH basis.

#### 2.5.3. Array Optimization based on an anatomically-constrained 3D sampling volume

In the third experiment we perform a single optimization run using an anatomically-constrained 3D sampling volume.

#### 2.5.4. Array optimization based on a 2D sampling volume

In this experiment we repeat the optimization experiment we performed on a 3D sampling volume, but this time on a 2D sampling volume of radius 15 cm. Note that 15 cm is the inner radius of the 3D sampling volume; however 2D volume-based optimization is not the same as the 3D optimization with sensor locations restricted to a 2D surface. The two procedures use different fitness functions, since they have different sampling volumes.

## 3. Results

### 3.1. Investigation of uniformly-spaced radial arrays

**Figure 4.**
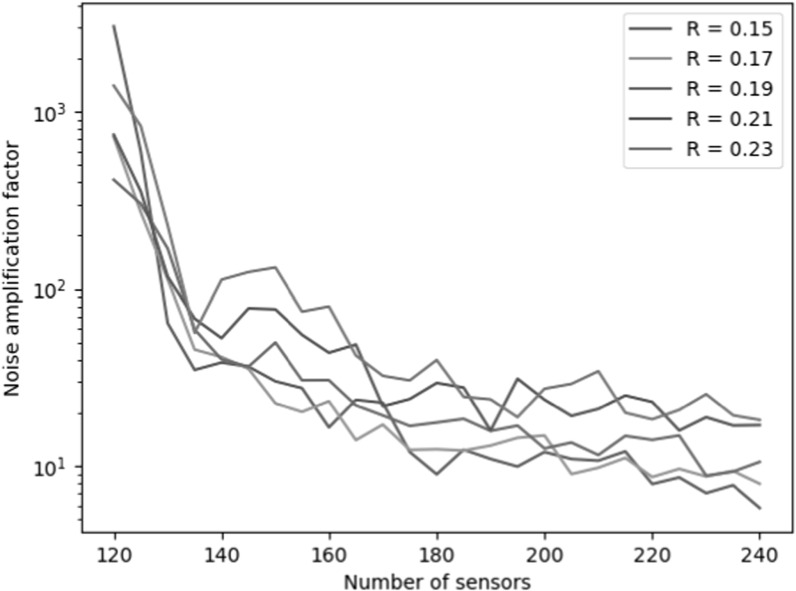
Behavior of the noise amplification factor for uniformly-spaced radial arrays as a function of the number of sensors and array radius *R*.

**Figure 5.**
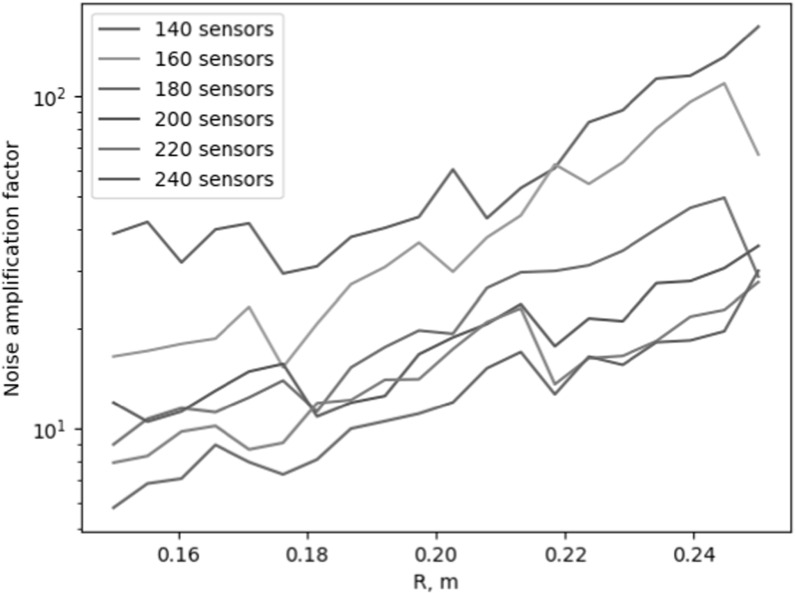
Behavior of the noise amplification factor for uniformly-spaced radial arrays as a function of the number of sensors and array radius *R*.

Figures 4 and 5 show the behavior of the noise amplification factor for uniformly-spaced radial arrays as a function of array radius *R* and the number of sensors. The noise amplification factor was calculated based on equation (12). From figure 4, we see that when the number of sensors is doubled from 120 to 240, the noise amplification factor shows a reduction of roughly two orders of magnitude. Figure 5 shows that the noise amplification factor improves when the array radius *R* decreases.

### 3.2. Array optimization based on a 3D sampling volume

**Figure 6.**
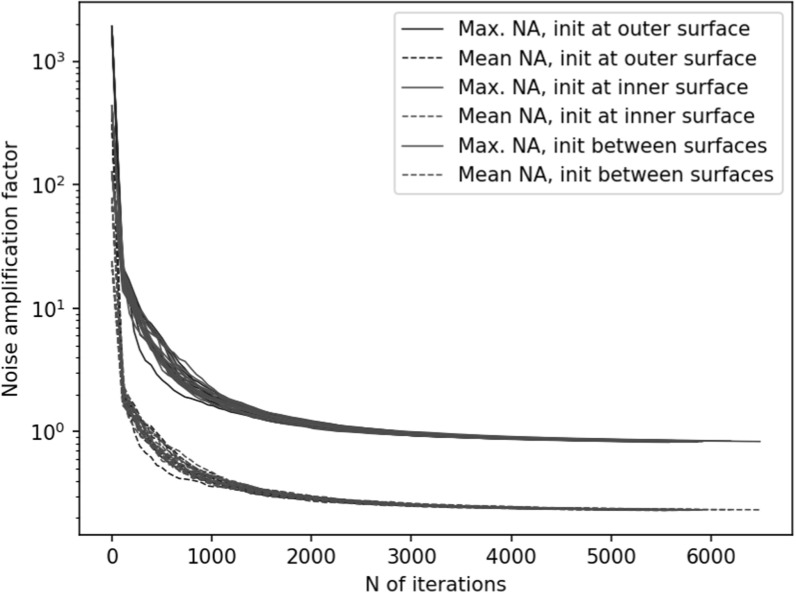
Noise amplification factor (NA) as a function of iteration during a 3D sampling volume-based optimization procedure. The optimization was repeated for different initial sensor locations: sensors on outer surface, inner surface, and halfway between the surfaces. *N* = 8 runs are plotted for each of these conditions. The solid and dashed lines indicate the maximum and mean noise amplification, respectively.

**Figure 7.**
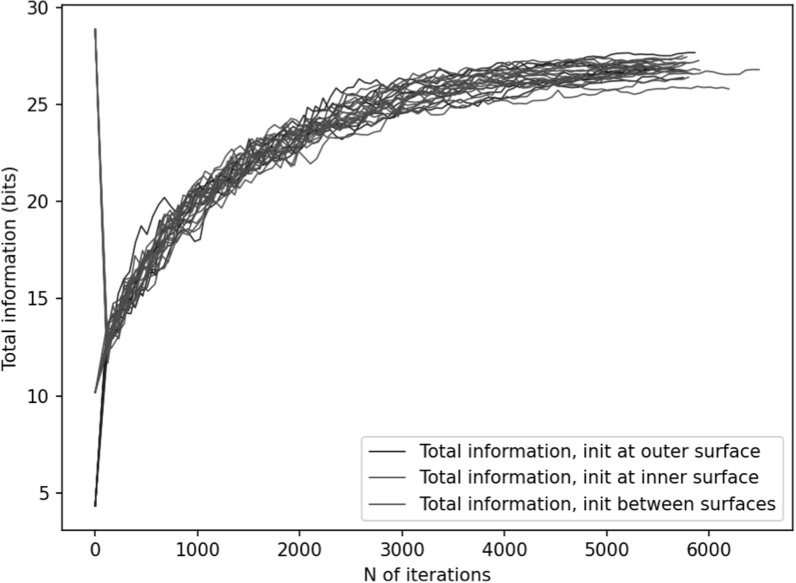
Total information as a function of iteration during a 3D sampling volume-based optimization procedure. The optimization was repeated for different initial sensor locations: sensor on outer surface, inner surface, and halfway between the surfaces. *N* = 8 runs are plotted for each of these conditions.

Figures 6 and 7 depict the behavior of the sensor array’s noise amplification factor and channel information capacity during a 3D sampling-volume-based optimization procedure. Both the noise amplification factor and the channel information capacity improve as more iterations are performed. The maximum and average noise amplification factors saturate approximately at values 1.0 and 0.2, respectively, while the channel information capacity reaches a value of approximately 30 bits per sample. The optimization was repeated with different initial sensor locations. In general, the results are quite consistent between runs, and the initial locations do not have a significant effect on the final optimization result.

**Figure 8.**
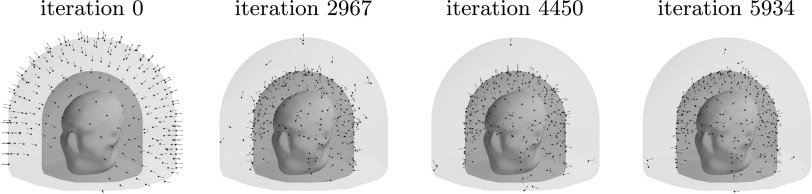
Progression of the sensor arrangement during the optimization for a 3D sampling volume.

Figure 8 depicts the evolution of the sensor array geometry during one optimization run, where the initial location of the sensors is on the outer surface. As the algorithm progresses, the sensors mostly migrate to the inner surface. On the average, about 6 sensors out of 240 remained close to the outer surface.

**Figure 9.**
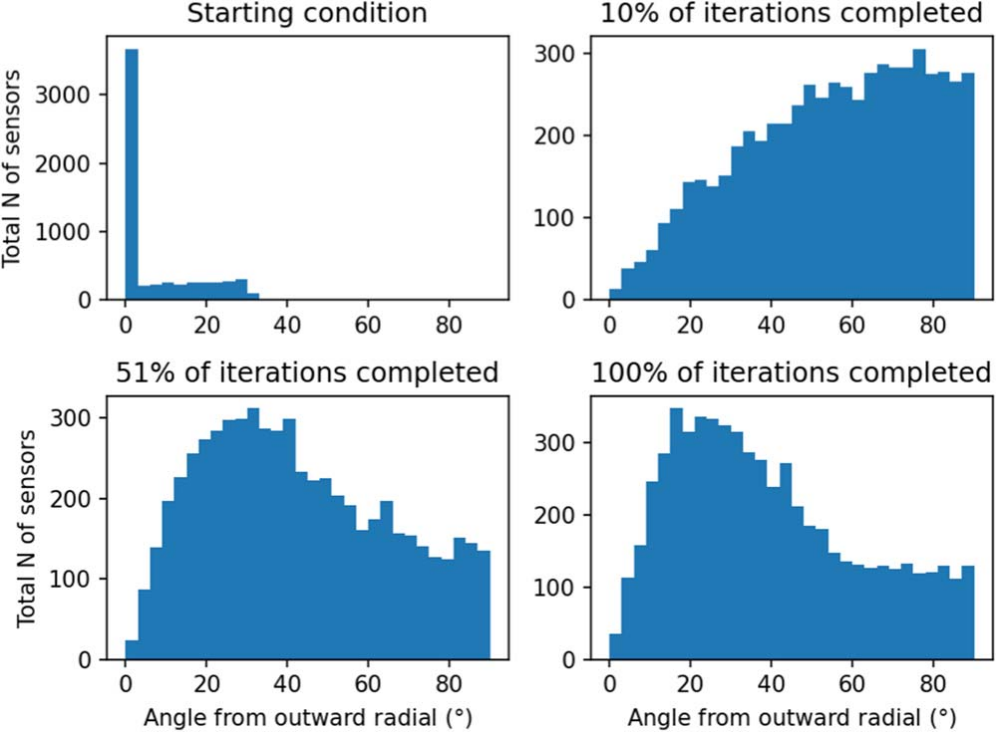
Distribution of sensor orientations during optimization. Data from *N* = 25 runs with the sensors starting at the outer surface are combined in the plot, for a total of 6000 sensors. ‘Outward radial’ refers to the radial normal of the spherical coordinate system, with the origin at the center of the sensor helmet.

Figure 9 illustrates the distribution of sensor orientations during optimization. In the initial condition, the orientations are mostly aligned with the radial normal of the spherical coordinate system; the alignment is not exact due to the helmet-like shape of the sensor array. At early stages of optimization, the tangential directions start to dominate. At convergence, the sensors have mixed orientations, with a majority of them being oriented more towards the radial direction.

**Figure 10.**
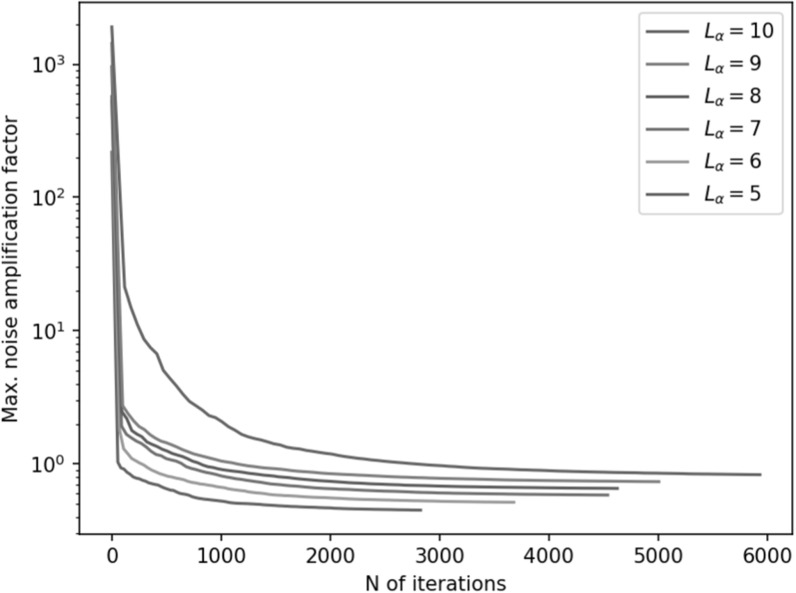
Maximum noise amplification factor (NA) as a function of iteration during a 3D sampling volume-based optimization procedure for different values of *L*_*α*_, with *L*_*β*_ = 3.

Finally, figure 10 illustrates the dependence on the method on the selected VSH degree cutoff. The optimization was repeated for different values of *L*_*α*_. Using a lower VSH degree cutoff results in faster convergence and lower overall noise amplification for the sensor array.

### 3.3. Array optimization based on an anatomically-constrained 3D sampling volume

**Figure 11.**
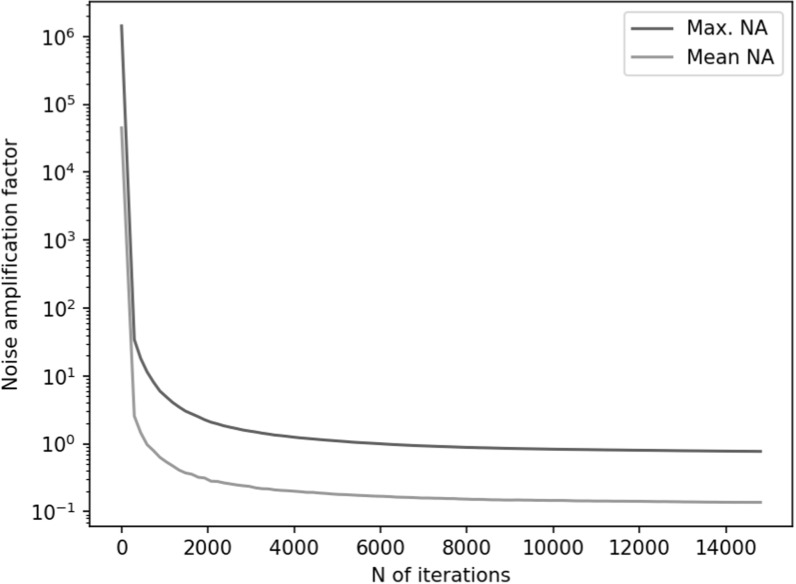
Noise amplification factor (NA) as a function of iteration for optimization on anatomically-constrained 3D sampling volume.

**Figure 12.**
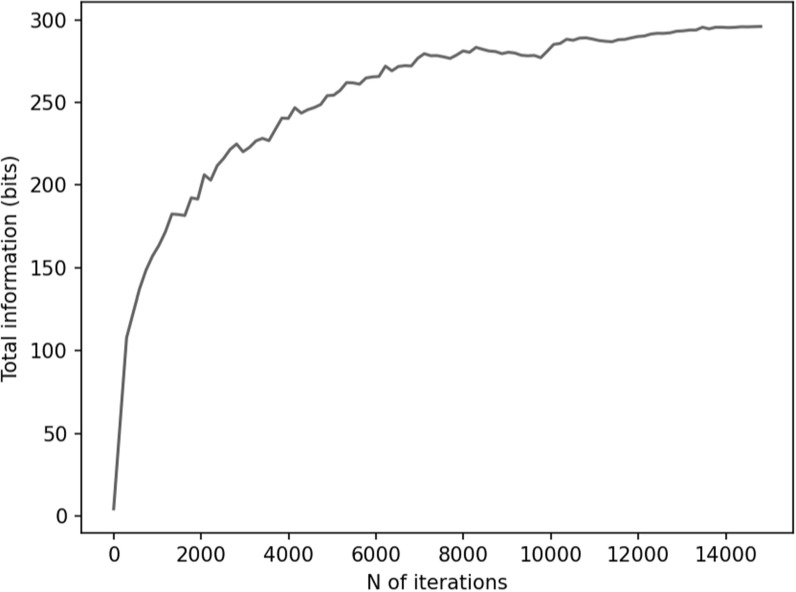
Channel information capacity as a function of iteration for optimization on anatomically-constrained 3D sampling volume.

Figures 11 and 12 depict the behavior of the sensor array’s noise amplification factor and channel information capacity during an optimization procedure for the anatomically-constrained 3D sampling volume. The optimization procedure generally behaves very similarly to that of the regular 3D sampling volume. One major difference is that the anatomically-constrained version attains much higher channel information capacity, which is to be expected, considering the fact that it can position sensors much closer to the sources of the signal inside the head.

**Figure 13.**
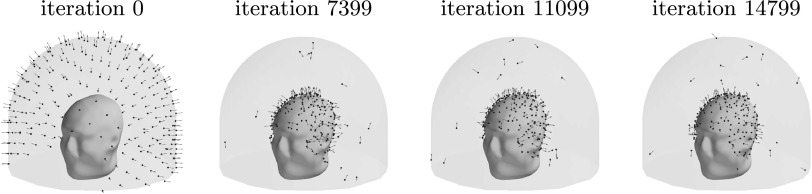
Progression of the sensor arrangement during the optimization for an anatomically-constrained 3D sampling volume.

Figure 13 depicts the evolution of the sensor array geometry during an optimization run. This too is qualitatively similar to the results for the regular 3D array.

### 3.4. Array optimization based on a 2D sampling volume

**Figure 14.**
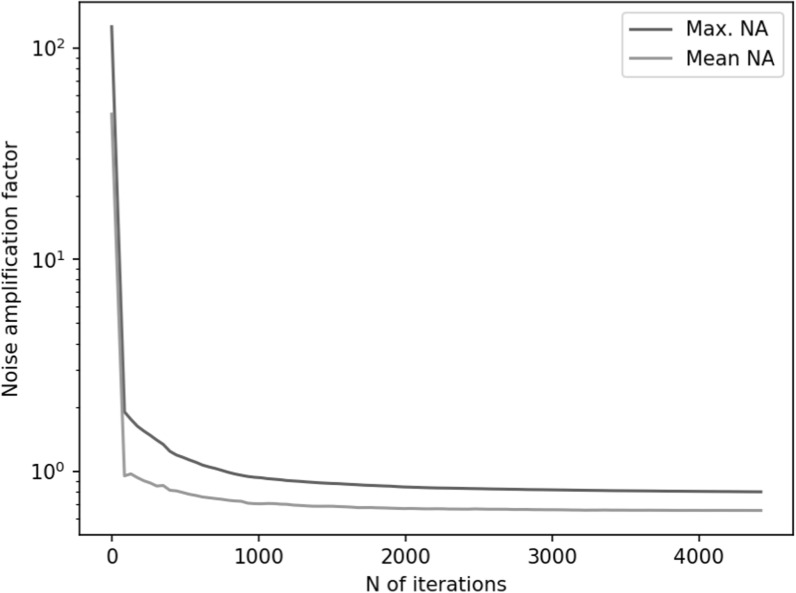
Noise amplification factor (NA) as a function of iteration for a 2D sampling volume-based optimization procedure.

**Figure 15.**
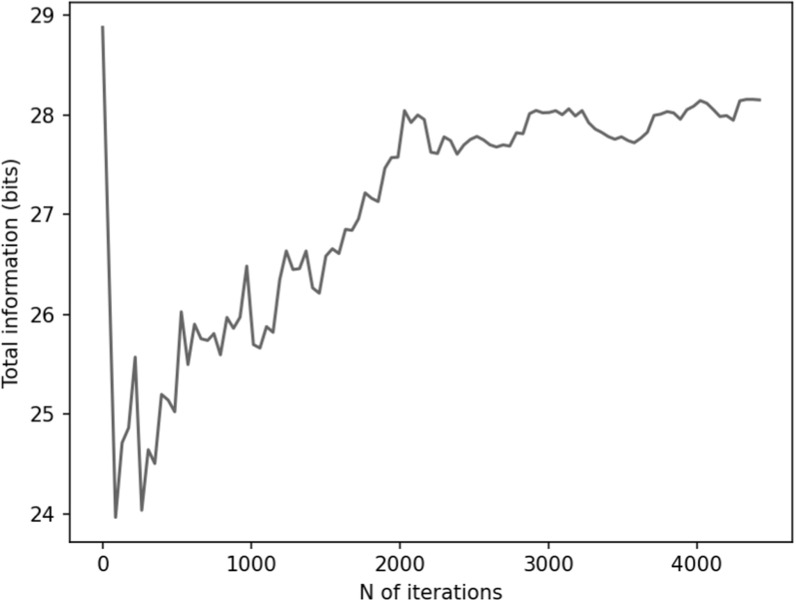
Channel information capacity as a function of iteration for a 2D sampling volume-based optimization procedure.

**Figure 16.**
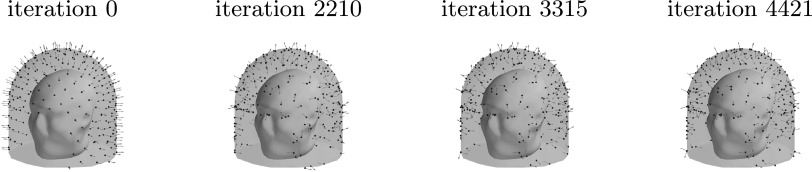
Progression of the sensor arrangements during the optimization for a 2D sampling volume.

In figures 14 and 15, we show the results for a 2D sampling volume, similarly to those presented in figures 6 and 7 for a 3D volume. Figure 16 depicts the behavior of the sensor array’s geometry during the optimization procedure. In this case, noise amplification factor improves as the algorithm progresses. Similarly, channel information capacity generally improves as a function of iteration. However, there is a steep drop in the channel information capacity after the first iteration. The algorithm starts with an initial configuration where the sensors are distributed uniformly on the surface and pointing radially (figure 16). After the first iteration, the algorithm deviates from this configuration and the channel information capacity decreases. However, as the algorithm progresses the channel information capacity eventually reaches the initial level while the noise amplification factor shows an improvement of approximately two orders of magnitude. Looking at figure 16, we observe significant changes in the sensor orientations, which correspond to an improved noise amplification factor and increased channel information capacity.

## 4. Discussion

### 4.1. General remarks

In this paper, we investigated the question of how to optimally measure the magnetic fields in MEG with a limited number of sensors while the assumptions about the underlying neural current distribution are minimal. As a general model for a discretized, multi-channel, magnetic field measurement we use the VSH expansion. This provides us with a a tool for interpolating the magnetic field within the sampling volume. The problem of interpolating magnetic field over a curl-free domain has been studied before. Solin *et al* (2018) suggest a method for interpolating magnetic field in an arbitrarily shaped curl-free region that relies on a scalar potential function in a way that is similar to what we do. However, operating over an arbitrarily-shaped domain, the method cannot utilize the approximate spherical symmetry of the measurement geometry that we have in the MEG case.

The VSH model can be thought of as a weakly informative prior to optimize the sensor positions and orientations. The VSH prior has three parameters: the origin and the cutoff values for the inner and outer VSH expansions. The origin represents the assumption about the sensor-to-source distance while the cutoff values of the inner and outer VSH expansions constitute an assumption that the magnetic field is bandlimited in the VSH (spatial-frequency) domain. As the VSH prior is general, i.e. it is not specific to any particular subject head geometry it can be used to construct a general optimized sensor array.

One unique feature of the VSH decomposition is that it allows us to separate the field to components representing the neural signals of interest and external interference components. By using both components to construct the field model (or prior) for sensor optimization, the sensor array simultaneously samples the neural signals and the interference allowing to separate them.

Compared to previous studies on MEG array optimization using fixed sensor orientations (Beltrachini *et al* 2021, Iivanainen *et al* 2021), the VSH formalism allows us to also optimize the sensor orientations. The obtained results suggest that when the sensors are measuring a single field component the optimal sensor orientation is not always radial as would be suggested when directly comparing radial component to the two tangential components (e.g. Iivanainen *et al* 2017). The deviation from radial orientation can be mostly explained by two different factors. First, the orientation of the spatial covariance function of the bandlimited VSH model is not radial everywhere in the sampling volume. Second, the introduction of external VSH components to the model necessitates sampling of the tangential components to allow better separation of the inner and external components.

Recently, optically pumped magnetometers (OPMs) measuring two (Colombo *et al* 2016, Borna *et al* 2017) or all three components (Brookes *et al* 2021, Boto *et al* 2022) of the magnetic field have been developed. We did not optimize arrays comprising of triaxial or dual-axis OPMs, but we note that the methodology presented in the paper can be used to optimize such arrays.

A central point of our approach to defining the MEG sensor array’s figure-of-merit is separating the question ‘What can we say about intracranial currents from extracranial magnetic field measurements?’ From the question ‘How can we measure extracranial magnetic fields as accurately as possible?’. However, it is not clear how these questions are related. The sensor array may sample a high percentage of the field energy (∼99%, for example) giving a highly accurate reconstruction of the magnetic field, but the source estimation might benefit from additional sensors.

### 4.2. Interpretation and significance of the obtained results

As shown in figure 4, as we interpolate the magnetic field based on the spatially discretized measurement, the noise amplification factor decreases as the number of sensors increases. This is an intuitively obvious result, but figure 5 also indicates that decreasing the physical dimensions of the actual array results in a decreasing noise amplification factor. This can be understood by an increased density of spatial sampling as the sensors will be distributed across a smaller surface area.

In order to validate our approach against other metrics, we chose to compare the progression of the noise amplification factor to the channel information capacity, which is a commonly used quantity in the evaluation of MEG sensor arrays. We found that decreasing noise amplification factor during the progress of sensor array optimization was consistent with increasing total information, as shown in figures 6 and 7. As a result of the optimization procedure, the sensors are distributed across the inner surface of the sampling volume with widely different orientations, see figure 8. Similar results, with respect to the connection between noise amplification and channel information capacity as well as the sensor orientations, were obtained in the 2D case, as indicated in figures 14–16.

It is intuitively desirable to place the sensors as close as possible to the head with sensor normal pointing symmetrically, e.g. in the radial direction. However, for the purpose of distinction between the internal and external magnetic fields, it is beneficial to break the spherical measurement symmetry as much as possible, as suggested already by Nurminen *et al* (2013). This can be achieved by having the sensors be close to the head while the sensor orientations become widespread and randomly distributed. At the end of the optimization procedure leading to these random orientations, the corresponding channel capacity returns to the initial level as well.

### 4.3. Other remarks

Note that in the process of optimization noise magnification factor drops below 1. This means that with our sensor configuration we can estimate magnetic field *everywhere, including the sensor locations*, better than what we get by directly measuring it with a single sensor.

### 4.4. Limitations

Our results rely heavily on the assumption that the magnetic fields within the sampling volume can be accurately modeled with a truncated VSH expansion (see the supplementary material). This assumption has some potential problems:

1. In real MEG measurements the assumptions of the VSH expansion about the current geometry (three concentrical compartments) do not hold because the middle compartment includes a part of the participants body (neck, etc) and thus cannot be guaranteed to be current-free. Moreover, for on-scalp sensor arrays, a single sphere separating the sensor array and the head cannot be found.
2. Truncating VSH expansion naturally introduces truncation error. The truncation error decreases when we increase the cutoff orders for the internal and external parts of the expansion (*L*_*α*_ and *L*_*β*_ accordingly). It is not clear which cutoff values are sufficient; they depend on the SNR of the measurement.
3. The residual VSH components of the field outside the truncated VSH expansion will alias if they are above the noise level and if the sensor array does not provide sufficient oversampling of a given truncation.

Moreover, strictly speaking, the interpolation noise computation only accurately models the noise for a *single* ‘virtual’ sensor. If we use it to model noise for a virtual sensor array of multiple sensors, the noise modeling for each sensor will be accurate, but the noise in the virtual array will be correlated across sensors, thus the array performance will not be the same as that of a real physical array with equivalent sensor noise.

## Data Availability

The data that support the findings of this study are openly available at the following URL/DOI: https://doi.org/https://github.com/andreyzhd/MEGSim. Data will be available from 15 February 2023.
