# Supplementary material for: A minimum assumption approach to MEG sensor array design
Source: Phys Med Biol. Author manuscript; Available in PMC 2023 Sep 11. (PMC10481949; doi:10.1088/1361-6560/ace306)
Supplement: Supplementary material [file NIHMS1927056-supplement-Supplementary_material.pdf]

Approximating Neuromagnetic Fields with Vector Spherical Harmonics

supplementary material for the manuscript
"A Minimum Assumption Approach to MEG Sensor Array Design"

Andrey Zhdanov, Jussi Nurminen, Joonas Iivanainen, Samu Taulu

June 15, 2023

We make the following assumptions: all the currents of interest (brain currents) are localized within a sphere (the sphere models the head, so we call it the head sphere) surrounded by a current-free volume that includes the sampling volume as a subset. Additionally there are some distant current sources (environmental noise) located much further away from the center of the sphere than any of the sensors (see Figure 1). More formally, there exists a single origin $\mathbf{r}_\mathrm{o}$ and two radii $R_\mathrm{inner} < R_\mathrm{outer}$, such that:

- all the current sources of interest (the sources whose fields we want to measure) lie within the distance R_inner from $\mathbf{r}_\mathrm{o}$, in other words, within the volume $\{\mathbf{r} : |\mathbf{r} - \mathbf{r}_\mathrm{o}| < R_\mathrm{inner}\}$ (we call it *inner volume*)
- all the noise sources lie further than R_outer from the origin, in other words, within the volume $\{\mathbf{r} : |\mathbf{r} - \mathbf{r}_\mathrm{o}| > R_\mathrm{outer}\}$ (we call it *outer volume*)
- there are no current sources within the shell between R_inner and R_outer, in other words, within the volume $\{\mathbf{r} : R_\mathrm{inner} < |\mathbf{r} - \mathbf{r}_\mathrm{o}| < R_{\mathrm{outer}_{\text{¸}}}\}$ (we call it *the source-free volume*), and the sampling volume V_samp is located totally within this shell.

For the aforementioned setup, the magnetic field $\mathbf{B}(\mathbf{r})$ everywhere throughout the 3D space is the sum of the magnetic fields generated by the currents in the inner and the outer volumes:

$$\mathbf{B}(\mathbf{r}) = \mathbf{B}_\alpha(\mathbf{r}) + \mathbf{B}_\beta(\mathbf{r}) \qquad \forall \mathbf{r} \in \mathbb{R}^3, \tag{1}$$

where $\mathbf{B}_\alpha(\mathbf{r})$ is the neuronal magnetic field that we want to measure and $\mathbf{B}_\beta(\mathbf{r})$ is the environmental noise.

Considering the fact that the neural electromagnetic phenomena can be accurately modeled by the quasistatic approximation of Maxwell's equations (Hämäläinen et al., 1993), the magnetic field $\mathbf{B}(\mathbf{r})$ throughout the source-free sampling volume V_samp is curl-free

$$\nabla \times \mathbf{B}(\mathbf{r}) = 0 \tag{2}$$

where $\mathbf{r} \in V_\mathrm{samp}$ is the vector specifying location.[1] It has been demonstrated (Arfken et al., 2013; Jackson, 1998) that under the aforementioned conditions

[1] From here onwards we assume the applicability domain of all the equations to be the sampling volume $\mathbf{r} \in V_\mathrm{samp}$ unless explicitely stated otherwise.

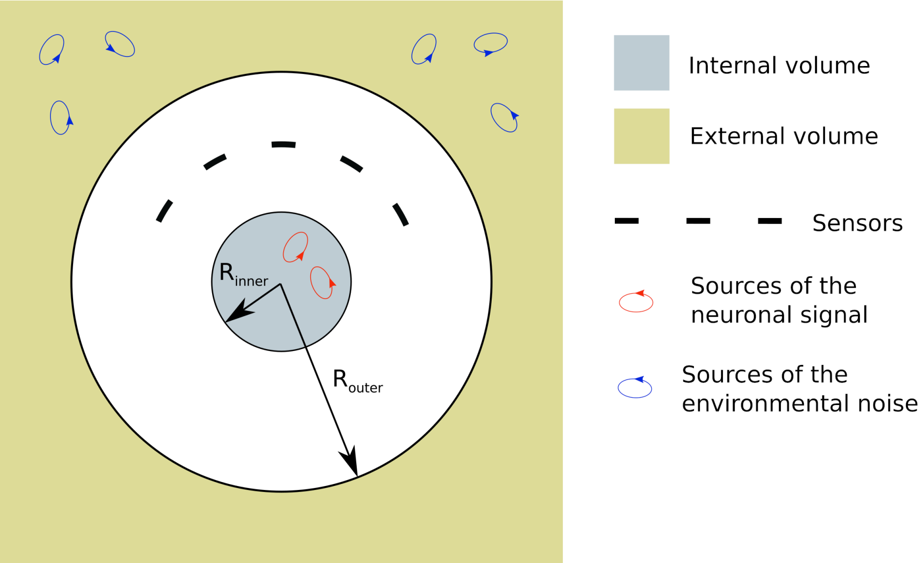

Figure 1: Single-origin VSH model

the magnetic fields $\mathbf{B}_\alpha(\mathbf{r})$ and $\mathbf{B}_\beta(\mathbf{r})$ can be expanded in terms of spherical harmonic basis functions:

$$\begin{aligned}\mathbf{B}_\alpha(\mathbf{r}) &= \sum_{l=1}^{\infty}\sum_{m=-l}^{l} \alpha_{lm}\mathbf{B}_{\alpha_{lm}}(\mathbf{r}) \\ \mathbf{B}_\beta(\mathbf{r}) &= \sum_{l=1}^{\infty}\sum_{m=-l}^{l} \beta_{lm}\mathbf{B}_{\beta_{lm}}(\mathbf{r}),\end{aligned} \tag{3}$$

where α_{lm} and β_{lm} are expansion coefficients, and $\mathbf{B}_{\alpha_{lm}}$ and $\mathbf{B}_{\beta_{lm}}$ are predefined basis functions that have the following form:

$$\begin{aligned}\mathbf{B}_{\alpha_{lm}} &= -\mu_0 r^{-(l+2)}\boldsymbol{\nu}_{lm}(\theta,\varphi) \\ \mathbf{B}_{\beta_{lm}} &= -\mu_0 r^{l-1}\boldsymbol{\omega}_{lm}(\theta,\varphi),\end{aligned} \tag{4}$$

where (r,θ,φ), is the representation of $\mathbf{r}$ in spherical coordinates centered at the center of the brain sphere, μ_0 is the magnetic permeability of vacuum, and $\boldsymbol{\nu}_{lm}(\theta,\varphi)$ and $\boldsymbol{\omega}_{lm}(\theta,\varphi)$ are the vector spherical harmonic functions[2]. Note that $\boldsymbol{\nu}_{lm}(\theta,\varphi)$ and $\boldsymbol{\omega}_{lm}(\theta,\varphi)$ do not depend on r. The set $\{\mathbf{B}_{\alpha_{lm}} : l = 0\ldots\infty, m = -l\ldots l\}$ is called the *internal* basis as it spans the space of all the magnetic fields originating form the currents in the inner volume. Similarly, the *external* basis $\{\mathbf{B}_{\beta_{lm}} : l = 0\ldots\infty, m = -l\ldots l\}$ spans the space of all the magnetic fields caused by the currents in the outer volume.

As the case with most infinite series expansions, the utility of Equation 3 comes from the fact that in real-world scenarios the magnitudes of α_{lm} and β_{lm} decrease pretty quickly with l. That is, in practice, we can restrict ourselves to a relatively small subset of all the basis functions corresponding to small values

[2]For the precise definitions of $\boldsymbol{\nu}_{lm}(\theta,\varphi)$ and $\boldsymbol{\omega}_{lm}(\theta,\varphi)$ see Taulu and Kajola, 2005.

of l without introducing any significant errors into the field estimate:

$$\begin{aligned}\mathbf{B}_\alpha(\mathbf{r}) &\approx \sum_{l=1}^{L_\alpha} \sum_{m=-l}^{l} \alpha_{lm} \mathbf{B}_{\alpha_{lm}}(\mathbf{r}) \\ \mathbf{B}_\beta(\mathbf{r}) &\approx \sum_{l=1}^{L_\beta} \sum_{m=-l}^{l} \beta_{lm} \mathbf{B}_{\beta_{lm}}(\mathbf{r})\end{aligned} \tag{5}$$

for some relatively small integers L_α and L_β.

The basis functions $\mathbf{B}_{\alpha_{lm}}(\mathbf{r})$ and $\mathbf{B}_{\beta_{lm}}(\mathbf{r})$ are perfectly known and do not depend on the intracranial source distributions or sensor array configuration. Hence if we know the values of a finite set of coefficients $\{\alpha_{lm} : 1 \leq l \leq L_\alpha, -l \leq m \leq l\} \cup \{\beta_{lm} : 1 \leq l \leq L_\beta, -l \leq m \leq l\}$, we know everything about the magnetic field in the sampling volume.

References

Arfken, G. B., Weber, H. J., & Harris, F. E. (2013). *Mathematical methods for physicists.* Elsevier.

Hämäläinen, M., Hari, R., Ilmoniemi, R. J., Knuutila, J., & Lounasmaa, O. V. (1993). Magnetoencephalography—theory, instrumentation, and applications to noninvasive studies of the working human brain. *Reviews of Modern Physics*, *65*(2), 413–497.

Jackson, J. D. (1998). *Classical electrodynamics.* WILEY.

Taulu, S., & Kajola, M. (2005). Presentation of electromagnetic multichannel data: The signal space separation method. *Journal of Applied Physics*, *97*(12), 124905.
